# Supplementary material for: Methylation of SPARCL1 Is Associated with Oncologic Outcome of Advanced Upper Urinary Tract Urothelial Carcinoma
Source: Int J Mol Sci. 2019 Apr 3;20(7):1653. doi: 10.3390/ijms20071653 (PMC6480388; doi:10.3390/ijms20071653)
Supplement: Supplementary file 1 [file ijms-20-01653-s001.zip › ijms-451070-sup R2 revision.docx]

**Supplementary Figures**


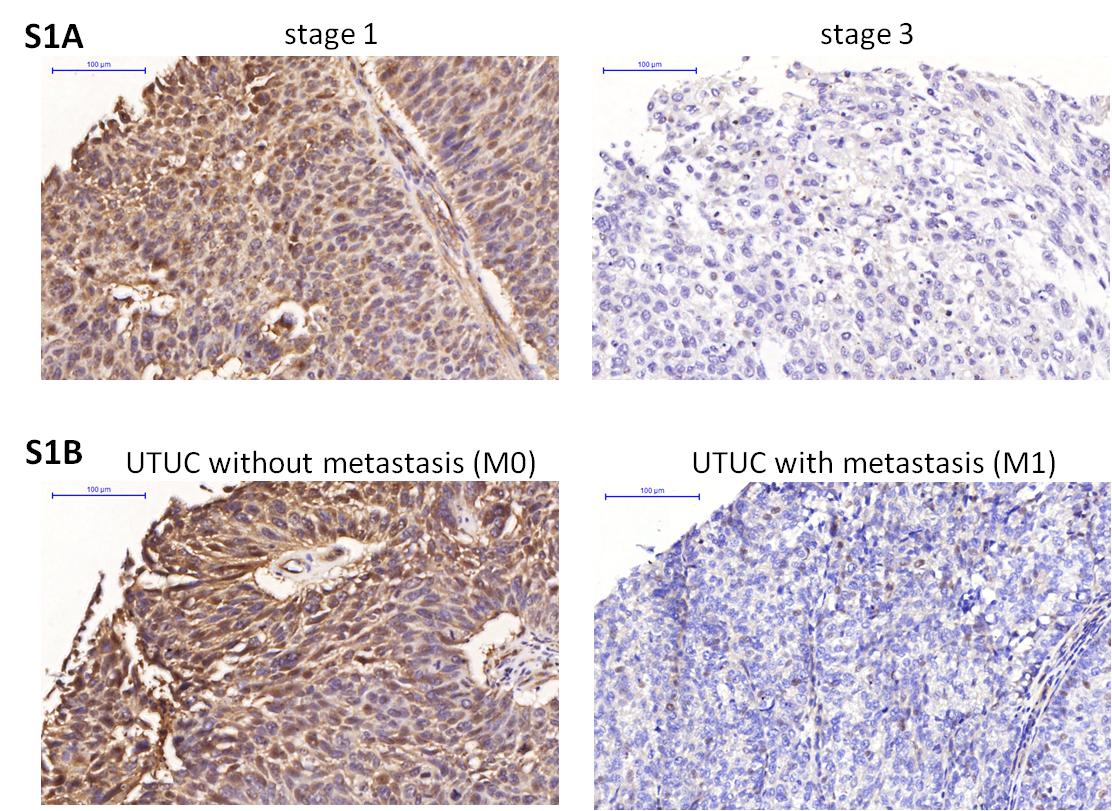


**Supplementary Figure 1.** Representative immunohistochemical staining of SPARCL1 protein in the upper urinary tract urothelial carcinoma. (**A**) Left, high expression of SPARCL1 in UTUC with pathologic tumour stage 1; right, negative expression of SPARCL1 in UTUC with pathologic tumour stage 3. Magnification: 200×. Scale bars, 100 μm. (**B**) Left, high expression of SPARCL1 in UTUC without metastasis (M0); right, low expression of SPARCL1 in UTUC with metastasis (M1). Magnification: 200×. Scale bars, 100 μm. Immunohistochemical staining showed that SPARCL1 was mainly located in the cytoplasm.
